# Intimate partner violence against women in Southern Punjab, Pakistan: A phenomenological study

**DOI:** 10.1186/s12905-022-02095-0

**Published:** 2022-12-08

**Authors:** Tehmina Sattar, Saeed Ahmad, Muhammad Asim

**Affiliations:** 1 Department of Sociology, Bahauddin Zakariya University, Multan, Pakistan; 2 Department of Sociology and Anthropology, Utah State University, Logan, UT 84321 USA; 3 Department of Community Health Sciences, Aga Khan University, Stadium Road, PO Box 3500, Karachi, 74800 Pakistan

**Keywords:** Socio-cultural dynamics, Intimate partner violence, Rural sphere, Domestic violence, South Punjab, Pakistan

## Abstract

**Background:**

Intimate Partner Violence (IPV) refers to behavior by an intimate partner that can cause physical, sexual, or psychological harm; is a common global public health issue requiring immediate attention. IPV is the most common form of violence in rural areas of Punjab, Pakistan.

**Methods:**

This qualitative phenomenological study collected 46 in-depth interviews from married women who experienced IPV in the rural areas of South Punjab. A semi-structured interview guide was used for data collection. These women were selected through a snowball sampling technique from October 2018 to March 2019. Researchers accessed the study setting with the help of gatekeepers (Lady Health Workers and Village Heads). The interviews were audio-recorded in the local language (Saraiki) and were translated into English. The data were analyzed using the thematic inductive analysis technique.

**Results:**

The study has presented multifaceted factors of IPV by using the socio-ecological framework in rural areas of South Punjab, Pakistan. The current study introduced culturally contextualized terminologies of "protection," "physical submissiveness," "mental delicacy," and "social security". For married women, culturally embedded terms became the primary cause of IPV. In addition, the study also highlighted some of the cultural terminologies (such as *run-mureed, watta*-s*atta, beghairat, izzat, *etc*.*) that are ubiquitous in the local context that sometimes intensifies IPV in the family and community sphere. Furthermore, the study discussed how gender-based inequalities trigger a status quo that ultimately creates power discrimination between spouses, which perpetuates violence in the domestic context.

**Conclusions:**

Gender-prejudiced roles and expectations imposed by orthodoxy, misinterpretations of Islamic teachings, and dominant patriarchy can be contested through awareness campaigns among the public, and gender sensitization drives among public institutions of police and judiciary. Education and employment-based can lead to women's empowerment and help to challenge the orthodox anti-feminist societal norms and the role of kinship-based networks in the family and community sphere.

**Supplementary Information:**

The online version contains supplementary material available at 10.1186/s12905-022-02095-0.

## Introduction

Intimate Partner Violence (IPV) is a common global public health issue requiring immediate attention [1]. According to UNESCO, 85% of the violence against women is perpetrated by their male intimate partners [2]. The World Health Organization (WHO) estimates that globally, one in three (30%) women experience violence from their partners [3]. IPV occurs in all settings and among socioeconomic, religious, and cultural groups [4]. However, the prevalence of and social and cultural context of IPV are more common in low-and-middle-income countries (LMICs) in the Middle East, South Asia, and Africa [5].

IPV has substantial consequences on women's physical, mental, and reproductive health [6, 7]. In addition, the social and cultural phenomenon of IPV is associated with individual factors such as socioeconomic, relationship status, and intergenerational exposure to domestic violence [8, 9], demographic factors such as age, number of children, family type, and risk of violence [10, 11], and socio-cultural factors such as alcohol or substance use, exposure to parental violence [12]. Younger women aged 15–19 years are at higher risk of IPV when compared with older groups of women. Similarly, women who have a lower level of education, lack of access to resources, and live in joint families are also at higher risk of IPV [13]. Furthermore, unplanned pregnancy [14, 15], inadequate social network support for the women [16–18], and family history of abuse place women at an increased risk of violence in the domestic spheres [19].

The prevalence of IPV against women in the domestic sphere has increased since the 1960s due to the complex role of women [20]. The lifetime prevalence of physical abuse experienced by women through their intimate partners ranges from 10% to more than 60% worldwide [21]. According to the national survey of Pakistan, 38% of women and 26% of men justify ‘wife beating' if a wife argues [22]. Strangely, women are more likely to justify IPV than men in Pakistan. The revealing fact about this social and cultural phenomenon is that 80% of households in Pakistan experience one or other forms of IPV. It is reported that 70% of Pakistani women are socialized to hide the situation of domestic violence as it will bring shame to their families [23, 24]. Moreover, women are trained by socio-cultural values to justify or accept IPV [25, 26].

There is a lack of direct access to the data on the socio-cultural dynamics of IPV in the domestic sphere of South Punjab. The region has rigid cultural values in which family structures are embedded. Most women lack control over domestic situations, and significant vulnerabilities establish the fertile ground for discordant power-sharing. The geographical locality of South Punjab has the cultural context of male supremacy where men have complete control over different household matters. With its unequal and hierarchical structures, patriarchal culture supports the men and their dominancy over the subordinated women.

The previous theoretical feminist regimes in the field have had a limited focus on the individual and cultural causes of IPV. The feminist analysis emphasizes the extensive efforts of gender and power in which the socio-cultural dynamics of IPV are discussed. The associated solution requires social and cultural transformation to promote women's empowerment and equality. The present research attempts to disentangle IPV from normative cultural patterns through women's lived experiences and perspectives of IPV and has documented the socio-ecological dynamics of IPV in rural areas of South Punjab, Pakistan.

## Methods

### Study design and setting

A qualitative phenomenological research design was used to carry out the current research. Phenomenology focuses on and describes the common meanings for several individuals of their lived experiences of a concept and social phenomenon [27, 28]. This research design was applied to explore the everyday experiences of married women who experienced IPV. Moreover, this design allows researchers to bracket or intentionally abandon their preconceived ideas, experiences, concepts, and theories from the phenomenon being studied [29].

**Fig. 1:**
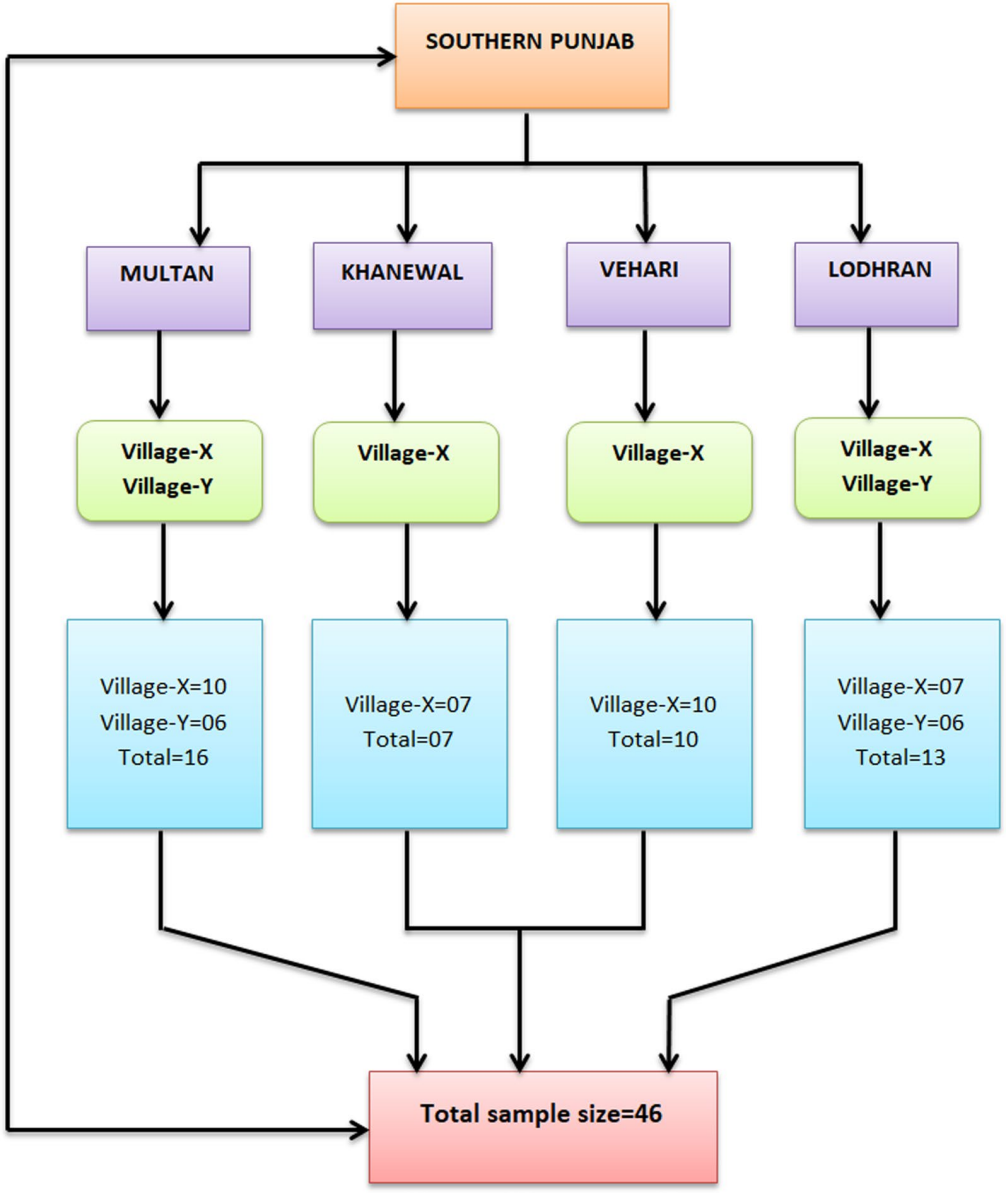
Procedure and sample selection from target population

Southern Punjab, Pakistan, comprises 11 districts, i.e., Multan, Dera Ghazi Khan, Layyah, Vehari, Bahawalpur, Rahim Yar Khan, Khanewal, Rajanpur, Lodhran, Muzaffargarh, and Bahawalnagar. This area comprises 32 percent of the population of Punjab province. The local language of this study area is Saraiki. A total of 46 in-depth interviews were conducted in 4 districts. Two villages were selected from Multan and Lodhran districts, while one was selected from Khanewal and Vehari districts. The detailed procedure and sample selection are mentioned in Fig. 1. The fact was that Multan and Lodhran districts are large in population density while Khanewal and Vehari are low in population size as compared to the other two districts. Therefore we selected two villages from the highly populated districts and one village from the low-population districts. The study vicinity comprised orthodox cultural values; therefore, we conducted in-depth interviews (IDIs) with the victimized battered women in the study setting [24, 30].

### Inclusion criteria, gatekeeping, and participants' requirement

The inclusion criteria for study participants included women who experienced IPV (i), were living with their spouse (ii), and agreed to participate voluntarily in this study (iii). To reach out to these women, the researchers sought the help of gatekeepers [Lady Health Workers (LHWs) from community health programs in their respective villages] to identify and recruit the participants. The close interaction with women in their catchment area (i), knowing the dynamics of family affinity (ii), and their easy access to each household (iii) were several reasons to approach the LHWs as the gatekeepers to recruit eligible participants. In addition, most families where women experienced IPV are known to the LHWs, who usually provide primary health care services at the household level. Therefore, LHWs were approached at the first stage to identify the households where women experience IPV. After identifying the concerned families, the LHWs facilitated arranging a meeting with the participants.

Consequently, a total of 63 households were identified by the LHWs, and 58 women were approached with the help of LHWs. During the meeting with the identified women, researchers screened 52 women who disclosed that they experienced IPV. Unfortunately, six women (11.53%) refused to participate in the study because they were scared of their spouses and were unwilling to reveal their household matters. Therefore, N = 46 women who experienced IPV and gave their consent to participate in the research were approached.

### Interview guide development

The authors developed a semi-structured interview guide for data collection (Additional file 1). Open-ended questions with several probing options were formulated by reading previous and existing relevant literature, meeting with gender specialists, and discussing with the research supervisor. During the interview guide development, the researchers focused on culturally specific terms (so that participants could attach themselves to the social world) for a profound understanding. For example, the term ‘Intimate Partner Violence' was used as, any act of physical and psychological violence including beating, kicking, slapping, coercive behavior, etc., perpetrated by the husbands. Before data collection, the interview guide was pilot tested on four women in the adjacent district. After pilot testing, questions related to sexual violence and sex-selected abortions were dropped due to being culturally sensitive, and the women were hesitant to answer these questions. The underlying reason for the exclusion of questions related to sexual violence was the prevailing patriarchy in rural areas. It was considered taboo to discuss the sexual matters of married women with some unknown person. Only in the case of some medical issue or some extreme marital disequilibrium, women can share such matters with other females or some religiously declared legitimized men (brother, father, husband, maternal uncle, and paternal uncle).

### Data collection

The process of data collection took place from October 2018 to March 2019. A total of N = 46 interviews were conducted with women exposed to IPV (see Table 1). The data were collected through face-to-face interviews in the Saraiki (local) language at women's homes in a particular place. This process allowed women to share their experiences of IPV without any fear or hesitation. The data was collected by the first author, due to her familiarity with the local culture, alongside two female researchers. To ensure privacy and confidentiality, the data was collected from the victimized participants in the absence of their abusive partners. LHWs provided us access to these participants as they were fully trusted by the family members, husbands, and victimized women.

**Table 1:** Socio-demographic profile of the victimized wives (n = 46)

Demographics of participants	Frequency	Percentage
Age		
Below 20 years	16	34.8
20–30 years	22	47.8
Above 30 years	08	17.4
Level of education		
Illiterate	19	41.4
Up to primary level	21	45.6
Up to secondary level	06	13.0
Family type		
Nuclear	12	26.1
Extended	25	54.3
Joint	09	19.6
Family size		
Less than five family members	14	30.4
5–10 family members	19	41.3
More than ten family members	13	28.3
Monthly household income		
Less than 15,000 PKR*	12	26.1
15,001–30,000 PKR	27	58.7
Above 30,001 PKR	07	15.2
Occupational status		
Housewife	15	32.7
Agricultural laborers	25	54.3
Government related job	06	13.0

Before the commencement of each interview, written consent was obtained to ensure their willingness to participate in the research, recording the interviews, and notetaking. All discussions were audio-recorded, and notes were also taken written by the research assistants to document the participant's verbal and non-verbal communication. The recorded data were immediately transcribed into English by the second author and were double-checked by the third author to ensure data authenticity. After each interview, a debriefing session was organized to identify preliminary coding and resolve discrepancies in the coding [31]. The in-depth interviews lasted between 60 and 90 min. The questions related to household conflicts like sexual and physical violence were asked during the transition stages of in-depth interviews. The data collection was halted once the saturation point was reached.

### Data analysis procedures

The data analysis process started with an intensive review of all transcripts and field notes through thematic analysis. The researchers used the concept of bracketing during the analysis of the data. All the preconceived ideas, personal observations, and reflexive notes were written by the authors before the data analysis [32]. The inductive method was used to identify significant themes [33]. This approach facilitated an intensive review of raw data to assemble the main themes, sub-themes, and interpretation of participants' responses. A comprehensive review of all transcripts prepared a preliminary list of themes. These preliminary themes were further classified into sub-themes. Through thematic analysis, themes were identified, and potential categories were coded with further classification according to the dimensional variations of the research. Analytical conduction and constant comparison strategies were also carried out by systematically examining the similarities between different dynamics related to IPV in the domestic sphere of the researched locale. An exhaustive list of themes was finalized that yielded a similar experience reported by all the participants. Moreover, overlapping themes were discarded from the final analysis after a profound discussion among researchers. Similar codes or recurring participant statements were omitted during manuscript preparation [34–36]. Moreover, the socio-ecological framework was used to present the findings into individual, relationship, community and societal elvel factors of IPV [37].

## Results

### Descriptive analysis

Table 1 highlights the demographic statistics of the respondents. In age, 34.8 percent were below 20 years, 47.8 percent were between 20 and 30 years, and 17.4 were above 30 years of age. In education, 41.4 percent of married women were illiterate, 45.6 percent had a primary level of education, and 13 percent had a secondary level of education. In family type, 26.1 percent lived in nuclear families, 54.3 percent lived in extended families, and 19.6 percent lived in joint families. In family size, 30.4 percent have less than five family members, 41.3 percent have 5–10 family members, and 28.3 have more than ten family members. In monthly household income, 26.1 percent have a monthly household income less than 15,000 PKR, 58.7 percent have a 15,001–30,000 PKR monthly household income, and 28.3 percent have a monthly household income above 30,001 PKR. Previous literature also mentioned the poverty level of South Punjab districts was 2.1% more than other districts of Punjab. The facts also demonstrated that 31% of the population of South Punjab lives below the national poverty line, while 55% live below half of Pakistan’s median per capita income [38]. Finally, in occupational status, 32.7 percent of married women were housewives, 54.3 percent were agricultural laborers, and 13.0 percent were doing some job.

### Thematic analysis

**Fig. 2:**
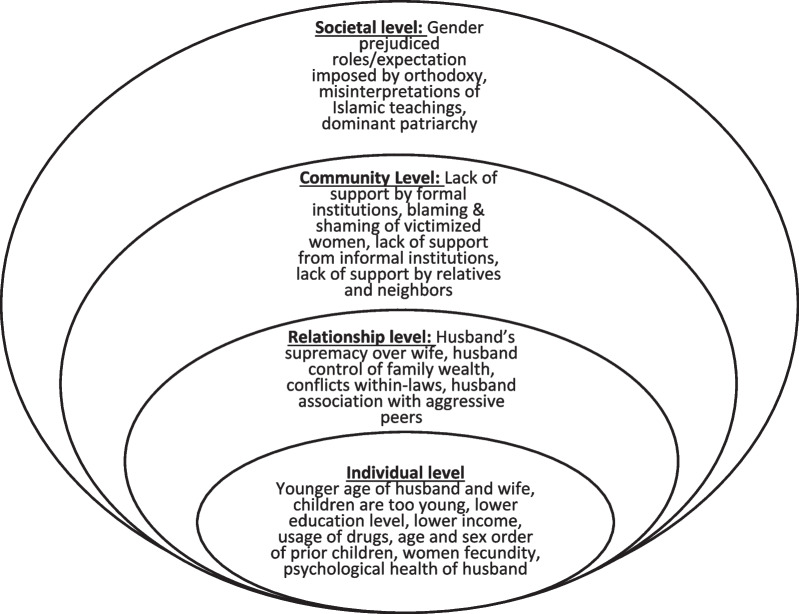
Socio-ecological model of IPV for the current study

The IDIs of women exposed to IPV were categorized into four major thematic levels: individual, interpersonal, community, and societal level factors by using a socio-ecological framework [39]. The major themes and sub-themes are summarized in Fig. 2.

#### Individual-level

The victimized women reported that individual factors were the significant demographic correlates that shaped their subjective experiences at interpersonal, community, and societal levels. The findings of the present study pointed out that the critical socio-cultural factors behind the prevalence of IPV at the individual level were (i) age of husband and wife (ii) lower education level of husband and wife (iii) lower income level of husband (iv) husband usage of mild drugs, alcohol consumption and engaging in gambling activities (v) sex order of prior children and fecundity of wife as well as (vi) psychological problems among husbands.

##### Age of husband and wife

The age of the husband and wife was the salient contextual factor that provoked IPV in the domestic sphere. The victimized women disclosed three situational age dynamics such as (i) when the husband and wife both were teenagers, (ii) when a wife was a teenager and the husband was more than ten years older than her, and (iii) when a wife was more than 30 years old and husband was younger than the wife (usually in cousin and *watta*-s*atta*^1^ marriages). Among the situations mentioned earlier, a higher level of IPV prevailed among couples in which both spouses were young, or when a husband was younger than the wife. For example, a victimized female reported that she was forcefully married to her 12 years younger cousin in a *watta-satta marriage* (exchange marriage) due to her increasing age, average beauty, and clumsy body. She shared her viewpoint with a saddened expression.

In my marital life, I think constant battering is a normal phenomenon because my husband was forced to get married to me.

In couples where husbands were older (at least ten years) than their wives became less exposed to IPV. The primary reasons were face beauty, objectified body consciousness, and fecundity of the younger wife. The most self-justifying phrases about the young wife were "younger bride will remain beautiful," "she will not get clumsy even after reproducing children," "she can reproduce more children due to her younger age," and "she can reproduce more sons due to her biological capacity to reproduce." Likewise, the findings revealed that IPV reduced with the increasing age of husband and wife as the children also grew older.

##### Lower education level of husband and wife

The lower education level of the husband and wife elicited IPV within the domestic milieu. The participants blamed how their parents intentionally kept them uneducated to subjugate their self-esteem to their husbands. Moreover, the study conceptualized that a higher level of education would be more destructive for wives due to the following reasons: cognizance of marital rights, awareness about reproductive rights, economic independence, and deliverance from forceful wedlock. To this connection, some participants revealed that education was perceived as *demaghi fatoor*^2^ (mental obsession or imperfection) for wives in a rural cultural context. Therefore, the low education level of a wife was used to countersign the husband's violent powers against a wife in wedlock. A 23 years old victimized participant validated these facts.

Since childhood, we (sisters) are socialized to stay away from education and to think about our marital life. Marital life means to comply with husbands and "say yes" to their mild and life-threatening violent acts.

Contrarily, the husband's low educational level was also dangerous for the wife. The victimized participants put forth the subsequent aftermaths of husbands' low education: lack of awareness about the marital rights of the wife, loss of civilized manners to maintain the peaceful marital environment at home, perceiving IPV as their fundamental right against their wife, execution of IPV as a symbol of male dominancy, belief that IPV is a mandatory act to maintain the status quo of patriarchy in wedlock and perpetration of IPV to satiate the manly expectations of society for the husband. Therefore, a lower education demonstrated a conflict between "*domination and subjugation of spouses*" in the domestic context.

##### The lower level of the husband's income

The findings revealed that women who experienced IPV had low household incomes. Financial hardship makes it hard to meet household utilities that, in turn, create tensions, frustrations, and quarrels between husband and wife. As wives were expected to tolerate marital disagreements and clashes, they had to surrender their arguments. Conversely, husbands vent their frustration on wives in the form of mild or brutal acts of violence. A young married bride who was beaten many times by her husband due to minor household-based financial problems shared her personal experiences.

Evidently, in the family sphere, the financial matters look like petty issues, but in fact, these are the real pre-conditions of IPV. Whenever my husband gets some extra money, then his mood swings, and I feel secure from his violent acts and abusive language.

Contrarily, the arguments of victimized women whose husbands were in better economic condition disclosed less exposure to IPV acts. For example, the husbands who owned some agricultural land had better income levels (i.e., more than 40,000 PKR), and they were less inclined towards IPV acts. Moreover, the working women supporting their husbands were also less exposed to IPV.

##### Husband's usage of drugs, alcohol consumption, and engaging in gambling activities

The victimized women reported that their husbands frequently use drugs and consume alcohol. Resultantly, they (husbands) lost their senses and used IPV against them (wives). These two allied traits were the "mannish personality of husband." Even mothers knew about the unethical activities of their sons. Still, they always justified it with the phrase "*man has every right*." Fourteen victimized women gave a consensual argument that their husbands frequently beat them due to alcohol consumption, and eight battered participants verified that their husbands did not frequently drink alcohol. Still, they were engaged in *parchi jua*^3^ (prize bond or number gambling). IPV sometimes becomes an inevitable social ramification whenever the husbands lose money in gambling. Endorsing this argument, a 27-year-old participant who was hospitalized after a severe battering reported that;

I was frequently battered by my husband as he was occasionally drunk at the gambling place. Once I asked him to avoid drinking and gambling, he has beaten me so severely that I was hospitalized for several days.

The battered participants also revealed that it was usual practice that the groom's friends arranged a bachelor's party on his wedding night. At this party, the married and unmarried men, along with the groom, consumed alcohol, showed off their weapons, and watched *mujra*^4^ (*a* dance party or performance by a woman or transgender*)*. This dance party was arranged to celebrate the groom's last night "as a bachelor." A newly married bride said she was beaten on the first wedding night because he was drunk and out of his senses. This argument was also endorsed in two interviews.

##### Sex order of prior children and future fecundity of wife

The participants reported that the previous sex order of the children and the future fecundity of the wife was the major adjoining IPV factors. The nexus for these two factors was the "*reproduction of sons*." Daughters signify social insecurity for parents, while sons have a higher place in rural social stratification. The pre-conditions of the sex order of children increase the probability of IPV, such as when the prior children had more daughters than sons, when the previous children had twin daughters, and when the previous children had one son and the last two children were daughters. A participant who was a frequent victim of violence gave a saddened expression and reported;

I had two sons and then twin daughters. I am frequently beaten by my husband because his expectation of twin sons was devastating.

The participants also disclosed how the total fecundity of the wife saved them from IPV. It guarantees the greater biological capacity to reproduce children which would consequently increase the probability of "*reproducing sons*." To endorse it, victimized females also gave a consensual argument that those women who had more sons than their prior children were less exposed to IPV. Moreover, the females who reproduced more daughters than sons but still have more than 15 reproductive years left (with an age of fewer than 30 years) were less likely to be exposed to IPV. The women also revealed that they could not visit doctors during pregnancy. The delivery of a child was also preferred through *dais*^5^ (traditional midwives) and LHWs. This method of childbirth increases the probability of miscarriages due to reproductive complexities. These issues shattered the fertility intentions of husbands about having more sons in their family, which ultimately may increase IPV.

##### Psychological problems of husband

Interviews with victimized women highlighted those specific psychological problems among husbands were also a major individual factor of IPV. The perception was that husbands are "*superhumans*" who have to protect the biologically inferior creature (the wife). In addition, anxiety, anger, and continuous family deterrence were the major psychological ailments among husbands. The women disclosed that the mental state of their husbands was designed by patriarchy, anti-feminism, and misogynistic ideology about women's existence in the household and societal sphere. A young woman with anger on her face shared her opinion;

This is the essence of our culture that the husband can get angry, can dominate, or can show contempt to his wife. These aspects are the masculine traits but known to be psychological problems of husbands.

The husbands’ childhood exposure to violence also gave acceptance, prevalence, and perpetuation of IPV in the domestic sphere. The childhood experiences of IPV have multi-dimensions consequences, such as considering IPV as an everyday life phenomenon. Men could use hegemonic powers over women through mild or severe acts of violence; a father is the role model of children; therefore, their acts of violence must be copied. Experience of violence is expected in the family sphere as it is embedded in traditional socialization codes, and IPV experiences in childhood reinforce the misogynistic ideology among men.

#### Relationship level (micro-level)

Micro-level factors constitute the emotional interactional patterns between individuals. The major sub-themes generated at the relationship level were husband supremacy over a wife, husband control of wealth in the family, fights, tensions, conflicts with in-laws, and association of the husband with aggressive peers.

##### Husband supremacy over wife

The victimized women unveiled that the husband was expected to be dictating, imperious and violent towards his wife in the marital relationship. This expectation was initiated by close relatives, household members, and *biradari*^6^ (caste and ethnic fellows). In this gender-based power nexus, the husband's supremacy was accepted as his fundamental marital right. The participants also communicated that the husband's domination was mandatory due to the following reasons: his being the bread earner of the family, protector of his wife, and provider of social security to his wife. Moreover, the husband was expected to maintain this gender inequality throughout his marital life. Otherwise, he would be labeled as *bivi thaly laga* or *run-mureed*^7^ (wife-subordinate) in the rural community. This gender-biased domination was vindicated due to physical submissiveness, emotional imbalance, and the psychological breakup of the wife. Validating this fact, a 43-year-old victimized participant reported;

My husband thinks that I am physically weak, tender, and emotionally malformed. Therefore, he frequently used violent acts to ensure his supremacy over me.

The husband's domination was a patriarchal tool to ensure female subjugation to his husband. This patriarchal tool was nurtured in the socialization codes of the husband from childhood. If a wife raised her voice against this superhuman dominance, she might suffer from marital disequilibrium, divorce, and separation.

##### Husband's control over wealth in the family

The husband's wealth control in the family was the primary factor for IPV against the wife. There were three major situational dynamics of spouse-earning activities, such as the husband was not earning, and the wife was earning (i), the husband was earning, and the wife was also earning (ii) and, the husband was earning, and wife was not earning (iii). Interviews highlight that the husband was the controlling agent of wealth in all the situations mentioned earlier because a husband is considered the protector of a wife who could take any verdict within the jurisdiction of the house based on decision-making autonomy. The normative orientation of the rural society exerts that the husband is physically, emotionally, and psychologically intense. Therefore he should take control of the wealth in the family. Endorsing this, battered women stated that the wife had to face severe violent consequences if she asked for "*participation in wealth control*" in the household sphere. A 35-year-old battered participant reported;

The major cause of physical battering is control of family wealth and resources. Wives cannot avoid these violent acts because they are completely deprived of participation in wealth control.

If a husband shared economic control with his wife, he was tagged as *beghairat*^8^ (lacking honor and respect). These denigrating titles also caused the husbands to become antagonistic and dominate their wives in economic matters. The rural community uses such tags as informal sources to maintain and control male dominance.

##### Fights, tensions, and conflicts with in-laws

Findings highlighted the role of *saas*^9^ (mother-in-law, usually the husband's mother) and *nand*^10^ (sister-in-law) were major aggravating agents of IPV. Contrarily, *susar*^11^ (father-in-law) and brothers-in-law were the least involved in violent acts against victimized women. The treatment of the mother-in-law with the newly married bride is humiliating, such as using offensive phrases of *paon ki juti*^12^ (worthless), *choti zaat di*^13^ (belonging to low caste), and *ganda khndan*^14^ (belonging to the lower family). The situation became worse when *saas* and *nand* formed an antagonistic group against victimized wives. This opposed group provoked IPV on various issues such as usage of modern methods of contraceptives, fertility intentions, spacing of children, giving birth to sons, isolation from decision-making in economic matters of household, avoiding visits to natal family, and avoiding visits to neighbors and friends. Validating the above findings, a 22-year-old victimized female argued:

My saas (mother-in-law) provoked fights and tensions in my marital life. My husband beat me several times as I argued with his mother on reproductive and economic rights in the family.

Some participants also reported that their *saas* (mother-in-law) triggered physical battering during their pregnancy. Consequently, it caused reproductive problems such as irregular periods, constant foul-smelling vaginal discharge, nausea, abdominal pain, uterus swelling, and even worse case miscarriages, etc. In addition, some participants reported that their *saas* (mother-in-law) was involved in forceful abortions on expecting the daughter.

##### Lack of support from relatives

Sometimes, the offensive behavior of police and courts compelled the victims to tolerate their husbands' violent acts. This culture of tolerance against IPV encourages the husband to execute violence on his wife with impunity. Participants revealed certain aftermaths of reporting IPV to police, such as husband, in-laws, and natal family becoming antagonistic, character assassination of the victim by police, and media involvement in these crime reports becomes a "*matter of shame*" for a victim as the community starts blaming them. A newly married bride described that when she tried to report these IPV acts to the police, she was scolded, abused, and beaten by her natal family. She further elaborated;

Police station is not a safe place for women to report the violent acts of their husbands. Actually, this is the place of further embarrassment and disgrace for victimized women.

The victim mostly avoided courts because the lawyers of the perpetrators confronted their character and marital integrity. The women set forth subsequent statements to demonstrate the non-supportive behavior of the judiciary, such as “wife is characterless," "wife has an affair with other men," "wife wanted to control the economic circle of the household," and "wife is modern and wants to live alone," and "wife is dishonoring the traditional community norms." These stigmatizations strengthened the perpetrator (husband) to degrade the victim of violence (wife).

### Community-level (Meso-level)

**Fig. 3:**
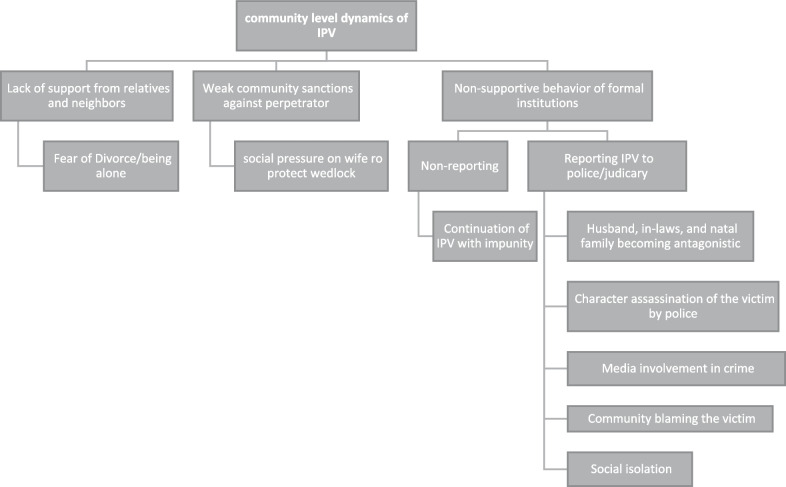
Community-level dynamics of IPV

The victimized women could not get support from the community due to gender-biased oppressive ideology. Non-supportive behavior of police and judiciary, social isolation, blaming and shaming of oppressed women, weak community sanctions against husbands, and lack of support from relatives and neighbors, etc., were major community-level factors of IPV. Community-level dynamics of IPV are summarized in Fig. 3.

#### Social isolation, blaming, and shaming of victimized women

Interviews highlight that community was conservative about women's placement in society. The married females who wanted to raise their voices against IPV acts were "interrogating their private marital life." Moreover, the punishment of "raising voice against the husband" deprives the victimized women of social support from natal family, in-laws, husband, neighborhood, etc. Consequently, these women were left alone with nobody to protect them in the male-dominating society. Furthermore, the victimized women felt "communally segregated" and "socially isolated." Thus, women preferred to tolerate the acts of IPV rather than face the severe aftermaths of "social cut-off." Validating these arguments, a newly married bride said;

I have to tolerate IPV acts as I want to save myself from social isolation. However, my husband knows that I cannot do anything to report IPV; therefore, he beats me frequently.

The participants disclosed that community members usually declared the husband "*innocent*" and victimized the wife as the "*cause of violence*." During data collection, we found that the list of reasons for blaming victimized women included charges that she was characterless, garrulous (*lambi zaban wali*), negating the husband's order, arguing with in-laws, taking frequent visits to natal family, and showing carelessness in domestic chores. This endless "blame game" compels participants to "hide," "tolerate," and "accept" the acts of IPV. In addition, the victimized women had to face bullying and shaming comments from the community members—these degradation modes revolved around humiliation, dishonor, scolding, and character assassination of the participants.

#### Weak community sanctions against husband

The community members considered the husband the "*sacred head*" of the household; therefore, he is free to execute IPV. Participants reported that when their neighbors intervened in their domestic and marital matters, but when the women asked for help against IPV, they did not provide it. During the transitional questions, the participants reported the subsequent phrases which demonstrated the weak sanctions for husbands in the community, such as beliefs that the husband is the owner of the family, the husband can make any decision in the household, the violence of the husband was for the betterment of the wife, slapping means mending the behavior of the wife, village dwellers will not accept women who stand against their husband, the wife should ignore the violent acts of husband for maintaining wedlock, and standing against their husbands will ruin the marital life of women. In agreement with these phrases, a 25-year-old participant who frequently became the victim of IPV validated;

Community norms expected that wife should be silent, abiding and tolerating towards husband's violent acts. Instead, these weak community sanctions encouraged my husband to perpetrate violence against me.

The participants reported that "*social pressure*" could be used as a solution to ensure that the "husband was also accountable for instigating IPV." Moreover, community members supported husbands for exerting IPV and at the same time disgraced their wives for being the victim of IPV.

#### Lack of support for relatives and neighborhood

The victimized females were deprived of social support from their natal family, in-laws, relatives, and neighborhood. The community thought that complaining about IPV would result in marital disequilibrium, separation, or divorce between the married couple. Resultantly, the women would be alone in society with nobody to protect them and their marital rights. Therefore the "fear of support deprivation" from village dwellers and "being alone" in the marriage was the major underlying causes of IPV (at the community level). The findings also unveiled that the neighborhood disclosed hatred and bullying against single, divorced, or separated women. The existence of the "married women" was considered to be the presence of their "husband." Endorsing these facts, a married female who faced and tolerated physical battering for the last seven years from her husband reported;

My husband knows that the community will not be supportive of me; therefore, he perpetuates violent acts against me.

#### Societal level (macro-level)

**Fig. 4:**
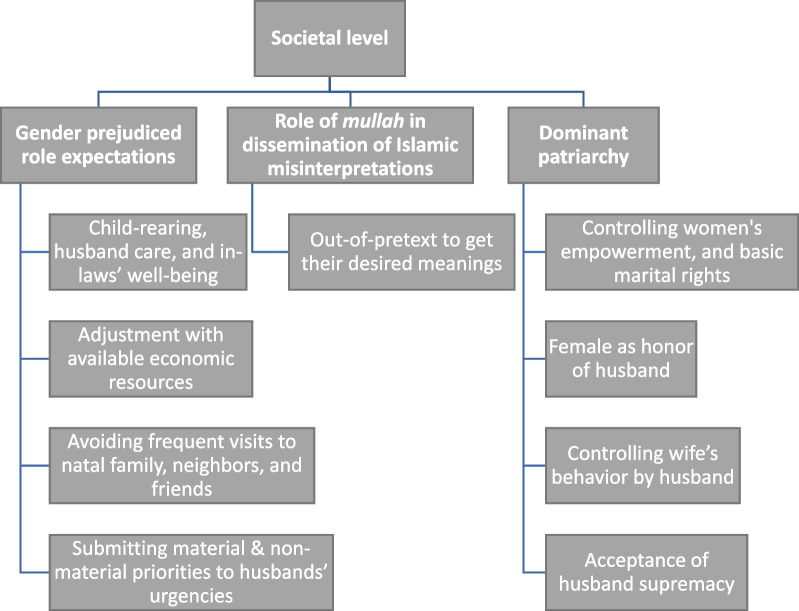
Society level dynamics of IPV

The victimized women blamed society for tolerance, acceptance, and approval of IPV. This culture of acceptance is a significant cause of violence perpetration at the societal level. These macro-level prerequisites of IPV have trickle-down effects on community, interpersonal and individual levels. Orthodox gender-prejudiced role expectations and impositions, the role of religious scholars in disseminating Islamic (mis)interpretations, and "dominant patriarchy" in marital relations were the major themes of IPV at the societal level. The societal level dynamics are summarized in Fig. 4.

##### Orthodox gender-prejudiced role expectations and impositions

The victimized women indicated the subsequent gender-prejudiced roles imposed upon married women, such as child-rearing, husband care, and in-laws' well-being. In addition, adjustment with available economic resources, avoiding frequent visits to natal family, neighbors, and friends, submitting material and non-material priorities to husbands' urgencies, and accepting husband supremacy were gender-prejudiced roles for married women. During the interviews, we found that gender-discriminated roles were imposed on women to maintain the male-dominated status quo. A victimized woman who was severely battered by her husband many times after marriage disclosed;

Society expected me that I should submit my ego, preferences, and wishes to my husband and fulfill the gender roles assigned to me by society. If I negate these gender roles, then surely I will face more violent acts from my husband.

##### Role of religious scholars in the dissemination of Islamic misinterpretations

The victimized women disclosed that the people of the study context gave utmost importance to Islamic teaching. The village dwellers took the guidelines of their daily problems from Islamic content. These religious teachings were taught, explained, and expounded by the religious scholars called "*imam*" and "*molvi*." Most men were involved in blind imitation and diffused religious ideologies. Many of the participants argued that *molvi* infused confusion in the minds of their husbands through misinterpretations of religious teachings. These misapprehensions of religious beliefs are out-of-pretext to get desired meanings. A battered female revealed that *imam masjid* (religious scholar of the mosque) preached that "Islam has allowed the husband to execute mild violent acts (such as slapping, and pushing, etc.) on wife." Moreover, the *imam* also said that the equality of spouses is a western concept that decreases the wife's dignity by making them equal to men. These arguments were endorsed by a 27-year-old victimized participant who said;

The imam masjid told my husband that the wife is a queen of the house. Therefore, the king must take care of his queen from social evils even by using some mild violent acts against her.

The participants gave a consensual argument that misinterpretation of Islamic teachings by religious scholars was the major macro-level factor of IPV. In addition, the participants revealed that they were beaten many times by their husbands for arguing over the preaching of religious leaders.

##### Dominant patriarchy

The participants used the phrase "dominant patriarchy" as the significant underlying societal factor of IPV. The data also highlighted that this phenomenon targeted women's independence, empowerment, and fundamental marital rights after marriage. A victimized married woman reported that dominant patriarchy in marital relations was related to dehumanization, incomplete identity, fragile body, and controlling wife's behavior by husband. These patriarchal beliefs created gender imbalances in marital relations which expected the wife to be submissive, obedient, and honoring (*izzat*) the husband. The participants used many phrases to show the perpetuation of IPV in the dominant patriarchy, such as my husband beats me because I am not allowed to visit my friends without his permission. Suppose my husband is scolding me and slapping me. In that case, it means he is protecting me from the notorious intentions of society, and my husband pressurized me for reproducing sons because it will make me socially secure. My husband can perpetuate violence to mend my behaviors because he knows right and wrong. A frequently beaten 33 years old participant endorsed these stated facts and argued;

I was socialized to tolerate the violence of my husband because he is head of the household. Now, I realized that male domination was designed to secure women in society.

The participants were antagonistic, exasperated, and annoyed by the spousal domination. Yet, contrarily, they were socialized and nurtured to accept this domination as a prerequisite of "protection from societal evils."

## Discussion

The present research used feminism as a significant theoretical explanation of IPV in the domestic sphere [40]. The findings reflected women's oppression in wedlock as a marital norm and the husband's right to his wife. These normative patterns were based on imbalanced power dynamics, prejudiced gender role expectations, and conventional social processes in wedlock [41]. The gender-biased anti-feminist perspectives explicated women to symbolize "honor" and "prestige" in Pakistan. For maintaining this traditional status quo, married females submitted fundamental marital rights to their husbands. Consequently, they confronted psychological oppression, emotional lapses, verbal abuse, and physical coercion from intimate partners [42, 43].

The feminist perspective used the words "wife battering," "spousal abuse," "family disequilibrium," and "marital violence" for enlightening IPV against women in wedlock. These theoretical debates elucidated that IPV is not a private matter of spouses, but it is a social-psychological phenomenon that is structurally embedded. In patriarchal societies, women tolerate IPV through various power-oriented tactics implied by their husbands [44]. The feminist perspective elucidated that women's subservient and compliant socialization restrained them in a complex configuration of anti-feminism and patriarchy. The empirical findings envisioned that the concept of a good wife is muted, sublimed, docile, compliant, and obedient to her husband. These gender asymmetrical roles were derived from male-dominating structures where a wife is expected to maintain marital harmony in the household through her self-sacrificing nature [45].

As evident, the present study used a socio-ecological model for explaining various dynamics of IPV in the contextualized study area. The current study's findings corroborated with the results of previous research that highlighted that brides who got married under the age of 20 years were at a greater risk of IPV [44, 46]. However, the young married brides were less likely to report IPV against them. The previous academic scholarship also indicated that the prevalence of IPV declines with the increasing age of women [47]. Previous literature illustrated the term “patriarchal bargain” which describes that women get greater autonomy and security within their marital bonds with increasing age [48–50]. As evident, the study context also showed that increasing the age of the women secured them from various misogynistic oppressive acts of their husbands. At this stage, these older women (in the relationship of mother-in-law) continue the cycle of violence against their daughter-in-law through patriarchal bargains [48].

Likewise, the other individual factor of IPV prevalence was the residence of married females. The women living in rural areas were more exposed to IPV than urban dwellers [43, 51, 52]. Among rural females, low education level was the reason for being the victim of IPV. The findings also showed that illiterate and less educated women were more exposed to IPV than women who were more educated. Moreover, the low education level of the husband also induced orthodox and patriarchal ideologies in their minds which triggered them to perpetrate violence against their wives. Similarly, previous empirical evidence also validated undesired pregnancies, the number of children, and the sex of children as the underlying cause of IPV [53]. In addition, the feminist perspectives reported that wives who faced constant acts of violence from husbands and in-laws became psychologically depressed, emotionally abused, and cognitively helpless [54, 55].

Previous studies validated the present research findings and indicated that the wife must submit her individuality to her husband and in-laws [56]. The primary reason was that husband supremacy was considered a fundamental marital right due to his protective and earning role. In extension, the gender-sensitive roles expected women to be submissive and muted in front of their husbands. The socialization codes of the wife inclined her to tolerate the violent acts and dominancy of the husband in the household sphere [5].

We also found that the other aligned factor behind IPV was fights, tensions, and conflicts with in-laws during data collection. Previous academic debates accredited that women were expected to be self-sacrificing and subjugated in front of in-laws [57, 58]. In Pakistani families, the newly married bride threatens her mother-in-law and sisters-in-law. These insecurities compelled the women to exploit the newly married bride and create conflicts and tensions between spouses. As the husband was in dominating position in marital relations, the result of these conflicts was violent acts and reproductive subordination in wedlock. The facts indicated that the power and control game, along with fear of dependency, creates a new form of patriarchy that is initiated and governed by older women (such as mother-in-law and sister-in-law) to protect their position and control in household matters [41, 42, 45].

In line with these relationship factors, the second wave of feminism explained that the perpetrator of violence (husband) possesses power and control over the victim (wife). These controlling supremacies created a domination-toleration relationship between perpetrators and victims in a male-dominated domestic environment [59]. Moreover, the aligned theoretical arguments in the "cycle of violence" also elucidated that victimized women remained in the cycle of violence in three major phases (i) tension building stage, (ii) the abusive relationship stage, and (iii) the honeymoon or forgiveness stage. The theoretical explanation divulged that the tension built in the marital relationship at the start of wedlock extends to a stage where a man cannot control and execute violence. Afterward, the perpetrator feels relaxed after venting his frustration and then apologizes to the victim for his violent behavior. Resultantly, the victim gave another chance to the perpetrator and forgot the perpetrator's violent acts. Then the honeymoon stage starts in which the victim thinks that the abuser will not execute the violent actions again. Constant exposure to violent acts resulted in feelings of helplessness, diminished decision-making, and the development of fear [41, 60].

As mentioned in the findings, women's exposure to IPV was more prevalent in the communities that adhered to the traditional patriarchal gender norms and beliefs. In this regard, a victim of IPV feared being blamed, shamed, and ridiculed by the police. The previous exploratory study also endorsed that the ridiculed behavior of police towards victimized women of IPV mostly prevailed in patriarchal societies [61]. Moreover, the police reports about IPV were further investigated and processed through the judiciary. Therefore, the victims must face problems and delays regarding assistance for IPV in law-enforcement agencies. The major reason was the criminal justice system is based on the stereotypes related to IPV [62, 63].

The present study's findings endorsed the socio-ecological model comprised of social structure, cultural beliefs, and network relationships to explain IPV prevalence in the study context [64, 65]. The previous theoretical framework validated that dominance and power were the major elements of patriarchy. These elements ensured male domination in society which trickled down to the household level [59]. The study context elucidated that those patriarchal norms inclined the husband to use their masculine powers to control their wives through various tactics ranging from psychological abuse to physical coercion [66]. Additionally, orthodox gender role expectations became the primary underlying factor towards IPV in Pakistan. These expectations put pressure on women to preserve family norms and traditional values by maintaining their "honor," "prestige," "submission towards the husband," "obeying in-laws," "confining them to household work," and "abiding by men's control over women" [41].

Aligning with the present research theme, a previous study conducted by Zakar et al. found that religious leaders advise married female to adopt the "forgive and forget approach" in wedlock. Under the prevailing patriarchal traditional norms, these religious leaders also blame the victim for initiating IPV by ignoring their marital responsibilities. These religious leaders misinterpret the Islamic teachings and attribute negative stereotypes to women such as "short-tempered" "emotional, and "physically docile" [5]. These religious leaders believe that women should be restricted by "the set of paternalistic stereotypes which confined them in restricted status" [67]. Feminism debates elucidated that patriarchal structure, orthodox gender role expectations, and extremist religious teachings prevailed in male-dominated societies. These dominating regimes compelled women to remain subordinate in front of their husbands. Different views of IPV can be attributed to the historical context of male power in the study area.

## Conclusion

In conclusion, husband supremacy and wife depreciation create gender inequality in marital relations. This inequality-based status quo creates power discrimination between spouses, which perpetuates violence in the domestic context. In an endorsement of these facts, we used a socio-ecological model to explore the underlying factors of IPV at four levels, i.e., individual, interpersonal, community, and society. The current study introduced culturally contextualized terminologies of "protection," "physical submissiveness," "mental delicacy," and "social security" for married women. These culturally embedded terms became the primary cause of IPV in the study vicinity. Maintaining marital stability and avoiding marital conflict was the wife's duty. Therefore, the ill-treated married females wanted to stabilize their marital life by fulfilling the inadmissible wishes of their husbands, in-laws, natal family, neighborhood, relatives, and community members. Endorsing these gender-biased oppressive norms and misogynistic behaviors of network relations, these victimized women accepted, tolerated, and supported these gender-based oppressive acts of violence and declared them as a "normal life phenomenon." The above-mentioned levels were used to maintain the patriarchal status quo, misogynistic ideology, and hegemonic masculinity in household and societal contexts. Moreover, the role of kinship-based networks in family and community and orthodox anti-feminist societal norms became the major prerequisites that generated the contextual factors for perpetrating IPV. Therefore, legislative measures could be adopted to challenge these gender-prejudiced hierarchies and ensure women's autonomy in the domestic and social fabric of Southern Punjab, Pakistan.

Due to a lack of time, resources, and ethical concerns, we could not interview the victimized women's judiciary, police, policymakers, and husbands. These participants can be included in future research to explore the underlying dynamics of IPV. The present study revolved dynamics of IPV within the family sphere. Future research can use the triangulation method within qualitative research be adopted, such as using obtrusive observations and grounded theory. The current study highlighted the perspective and lived experience of women, future research can collect the data from social activists, NGOs, and government officials to triangulate the data and to develop an intervention to create awareness among female and male members related to human rights, etc. The primary strength of the present study is to explore the causes of IPV at the individual, interpersonal, community, and societal levels. The other strength of this study is to elicit information from women who had lived experiences of intimate partner violence. This method was used to explore the opinions of victimized women about their personal experiences of IPV.

### Strengths and limitations

The primary strength of the study demonstrated the underlying causes of IPV at the individual, interpersonal, community, and societal levels. Furthermore, his study explored the experiences of women who were victims of domestic violence to capture their lived experiences. The major limitation of the study was to collect the data through in-depth interviews. The study revolved dynamics of IPV within the family sphere.

### Recommendations and future policy implications

Based on the findings of the present research, we recommended that government should spread community-level awareness programs to sensitize the general public about the marital rights of women to overcome violent relationships. Moreover, husbands can be the major agents of change by avoiding IPV in marital relationships. Therefore young adolescent boys should be socialized to maintain their marital relationship through tolerance, respect, intimacy, and esteem. Despite this, education and employment for women can be helpful to an elevated and respected position within the household and community level ultimately changing the attitude towards IPV at the individual, interpersonal, and community levels.

## Supplementary Information

**Additional file 1**. Semi-structured interview guide of the study.

## Data Availability

The datasets generated and/or analyzed during the current study are not publicly available due to the confidentiality of the respondents. Since women shared their personal experiences related to intimate partner violence and the data contain the identification of study participants but are available from the corresponding author on reasonable request.

## Footnotes

1 A custom involving simultaneous exchange marriages in a household.

2 The literal meaning is mental imperfection or obsession; this connotation is used for women's education to snub it.

3 a form of gambling in which number is used. a parchi with seven numbers is sold. In the draw usually held every fortnight, if the first prize winning number is precisely matched with one's parchi number, they get a big prize from the dealer. Parchi holder gets rewards on match first, second, and so on digits of the parchi.

4 a dance party or performance usually by women or transgender in large or private gatherings. It emerged during Mughal rule in the sub-continent, but stage drama culture had made mujra format more popular.

5 A traditional midwife is an indigenous method of giving birth to the child. It is still common in low-income and rural areas.

6 It is an extended association of relationships among people based on caste.

7 The stigmatization of husbands who love and treat their wives fairly. '*bivi thaly laga*' is also used in the same connotation. The literal meaning of this word is subordinate to the wife.

8 An offensive and abusive term with literal meaning as lacking honor or respect.

9 In the relational politics in family sphere, refers to the mother of husband, usually translated as mother-in-law.

10 nand is the sister in law, and.

11 susar is the father-in-law for the wife from the husband's side.

12 These are the taunts and offensive phrases used in fighst within family sphere in rural area. *paon ki jutti* has a literal meaning of footwear that refeclts being worthless,

13 belonging to a low caste family, and.

14 belonging to lower family.
