# Supplementary material for: Intimate partner violence against women in Southern Punjab, Pakistan: A phenomenological study
Source: BMC Womens Health. 2022 Dec 8;22:505. doi: 10.1186/s12905-022-02095-0 (PMC9730583; doi:10.1186/s12905-022-02095-0)
Supplement: Supplementary file 1 — Additional file 1. Semi-structured interview guide of the study. [file 12905_2022_2095_MOESM1_ESM.docx]

**INTERVIEW GUIDE**

**Demographics of the victimized married females:**

- Participant Name
- Age
- Education level
- Family income
- Occupation
- Residential status

**Socio-cultural dynamics of IPV:**

1. How will you define your married life?
2. What is the status of husband in the household?
3. What is the status of mother-in-law in the household?
4. What is the status of natal family in mediating the marital issues of the married couple?
5. What is the status of in-laws in mediating/instigating the marital relations of the married couples?
6. Do you agree that problematic married life will leads towards IPV or vice versa?
7. Are you in favor of mild IPV?
8. Do you think that IPV is justified in Islamic religion?
9. Do you think that IPV is justified in socio-cultural context?
10. How your marital solidarity changes after perpetuation of IPV?
11. Report the most frequent causes of IPV in your household context?
12. Report the intensified causes of IPV in your marital relationships?
13. Do you think that patriarchy can play a pivotal role in instigating IPV against women?
14. Do you think that male supremacy is the root cause of women subordination?
15. Do you that husband impose their masculine powers to dominate over wife?
16. Do you think that women have the right about their decision making power over fertility intensions?
17. What is the role of cultural context in ensuring husband dominancy over women?
18. How the husband underestimate their wife in a marital relationship?
19. What are the major modes of violence perpetuation by the husband towards its wife?
20. What are the major responsibilities of husband that gave them elevated status than their wife?
21. Do you think that the emotional state of wife is considered to be the major prerequisite for IPV against them?
22. What is the role of in-laws in perpetuating violence against women?
23. Do you think that the husband is loyal to his own family members and become antagonistic towards their wife?
24. Do you think that violence is initiated by in-laws and perpetuated by husband?
25. Do you think that the elevated status of husband is given by household and society?
26. What is the vision of religious leaders about perpetuating violence?
27. What is the role of religious leaders about the role of husband in household?
28. Do you think that religious leaders are correctly interpreting the Islamic rules about IPV?
29. It is general perception that religious leaders are actually cultural puppets who are encouraging the husbands to use violent acts against their wives? What is your viewpoint about this?
30. What is the stance of religious leaders about the protective role of husbands for their wife?
31. In your viewpoint, how the religious leaders attributed the domestic violence to be the religious right of men?
32. How religious leaders targeted the Western agendas to intervene in exaggerating the concept of violence against women?
33. Do you think that women autonomy is considered to be the sin in religious context? Also explain that how religious leaders interpreted this?
34. In your viewpoint, in-laws are more prorogating towards violence or natal family?
35. What are the major in-laws relations that are involved in perpetuating violence?
36. How mother-in-law plays a pivotal role in propagating violence against women?
37. Do you think that mother-in-law is the psychological abuser of the victimized females?
38. Do you think that mother-in-law is the verbal abuser of the victimized females?
39. Do you think that mother-in-law I the physical abuser of the victimized females?
40. Do you think that victimized women become exposed to violence because they are not supported by their closed network relations?
41. Do you think that husband is the direct source of perpetuating violence?
42. What is the status of a man who takes care of his wife and not involved in violent acts?
43. What is the status of man who perpetuates violence against women?
44. Do you agree that verbal violence has no contextualization in the study vicinity?
45. Do you think that psychological violence has no contextualization in the study vicinity?
46. Do you agree that physical violence is considered to be the normal life phenomenon in the study context?
47. Explain that how the physical violence is related with the reproductive health of the married females?
48. Explain the major adverse health effects of physical violence on reproductive health of the married females?
49. Do you think that after the execution of violence, natal family becomes the major support system for the victimized women?
50. Do you agree that the acts of violence increases against pregnant women as compared to the non-pregnant women?
51. What is your point of view about the role of natal family after the perpetuation of violent acts against women?
52. Do you think that natal family becomes silent on IPV to safe the married life of their daughters?
53. Do you think that natal family can actually become successful in revolving the conflicts between husband and wife?
54. Do you think that natal family can protect the victimized women from further violent acts?
55. Who is the most supportive person in natal family for the victimized women?
56. What are the major issues in which your natal family members supported you (in antagonism with the viewpoints of in-laws)?
57. What is your viewpoint about the social support provided to the husbands for justifying their violent acts?
58. Do you think that the religion provides the right to the husband to beat his wife?
59. Do you that the social structure provides the rights to husband to beat their wife?
60. Do you agree that victimized women themselves become the major supporters of violent acts against them?
61. Do you think that the causes of IPV are directly related with the aftermaths of IPV?
62. Do you think that IPV can deteriorate the peaceful environment of the marital life?
63. Report the major physical aftermaths of IPV with victimized women?
64. Report the reproductive aftermaths of IPV with victimized women?
65. Report the major socio-cultural issues related with IPV with victimized women?
66. Report the psychological aftermaths of IPV with victimized women?
